# Plasminogen and the Plasminogen Receptor, Plg-R_KT_, Regulate Macrophage Phenotypic, and Functional Changes

**DOI:** 10.3389/fimmu.2019.01458

**Published:** 2019-06-28

**Authors:** Juliana P. Vago, Michelle A. Sugimoto, Kátia M. Lima, Graziele L. Negreiros-Lima, Nagyung Baik, Mauro M. Teixeira, Mauro Perretti, Robert J. Parmer, Lindsey A. Miles, Lirlândia P. Sousa

**Affiliations:** ^1^Department of Molecular Medicine, The Scripps Research Institute, La Jolla, CA, United States; ^2^Center for Drug Research and Development, Institute of Biological Sciences, Federal University of Minas Gerais, Belo Horizonte, Brazil; ^3^Barts and The London School of Medicine, William Harvey Research Institute, Queen Mary University of London, London, United Kingdom; ^4^Department of Clinical and Toxicological Analyses, School of Pharmacy, Federal University of Minas Gerais, Belo Horizonte, Brazil; ^5^Department of Medicine, Veterans Administration San Diego Healthcare System, University of California, San Diego, San Diego, CA, United States

**Keywords:** resolution of inflammation, plasminogen system, plasminogen receptor KT, macrophages reprogramming, efferocytosis

## Abstract

Inflammation resolution is an active process that functions to restore tissue homeostasis. Clearance of apoptotic leukocytes by efferocytosis at inflammatory sites plays an important role in inflammation resolution and induces remarkable macrophage phenotypic and functional changes. Here, we investigated the effects of deletion of either plasminogen (Plg) or the Plg receptor, Plg-R_KT_, on the resolution of inflammation. In a murine model of pleurisy, the numbers of total mononuclear cells recruited to the pleural cavity were significantly decreased in both Plg^−/−^ and Plg-R_KT_^−/−^ mice, a response associated with decreased levels of the chemokine CCL2 in pleural exudates. Increased percentages of M1-like macrophages were determined in pleural lavages of Plg^−/−^ and Plg-R_KT_^−/−^ mice without significant changes in M2-like macrophage percentages. *In vitro*, Plg and plasmin (Pla) increased CD206/Arginase-1 expression and the levels of IL-10/TGF-β (M2 markers) while decreasing IFN/LPS-induced M1 markers in murine bone-marrow-derived macrophages (BMDMs) and human macrophages. Furthermore, IL4-induced M2-like polarization was defective in BMDMs from both Plg^−/−^ and Plg-R_KT_^−/−^ mice. Mechanistically, Plg and Pla induced transient STAT3 phosphorylation, which was decreased in Plg^−/−^ and Plg-R_KT_^−/−^ BMDMs after IL-4 or IL-10 stimulation. The extents of expression of CD206 and Annexin A1 (important for clearance of apoptotic cells) were reduced in Plg^−/−^ and Plg-R_KT_^−/−^ macrophage populations, which exhibited decreased phagocytosis of apoptotic neutrophils (efferocytosis) *in vivo* and *in vitro*. Taken together, these results suggest that Plg and its receptor, Plg-R_KT_, regulate macrophage polarization and efferocytosis, as key contributors to the resolution of inflammation.

## Introduction

Inflammation is the physiological response of the host to infectious or sterile injurious stimuli that ensures rapid and successful restoration of the tissue, and requires production of mediators and activation of signaling pathways (1). Anti-inflammatory/proresolving mechanisms are driven by a complex set of mediators that regulate cellular events required to clear inflammatory cells from sites of injury as a prerequisite for restoration of homeostasis (1–4). Phagocytic clearance of apoptotic cells (efferocytosis) at sites of inflammation also plays an important role in the resolution of inflammation by inducing remarkable macrophage phenotypic plasticity (5). Classically activated macrophages, M1 macrophages, are pro-inflammatory and enable host defense against infection, while M2 macrophages (alternatively activated macrophages) display anti-inflammatory and tissue remodeling properties, and play a key role in the resolution of inflammation (6, 7).

Plasminogen (Plg) is a zymogen (synthesized in liver) that subsequently is activated by plasminogen activators to generate plasmin (Pla), the major enzyme responsible for fibrin clot degradation *in vivo* (8, 9). In addition to fibrinolysis, the Plg/Pla system regulates the recruitment of mononuclear cells during the inflammatory response (9–12) and in non-phlogistic settings (13). The Plg receptor, Plg-R_KT_, is a unique transmembrane receptor with a C-terminal lysine exposed on the cell surface that interacts with Plg to promote Plg activation (14–16). Plg-R_KT_ accounts for the majority of the Plg binding capacity of macrophages (17) and regulates macrophage recruitment in a model of sterile peritonitis (16).

While the functional role of the Plg/Pla system in the pro-inflammatory phase is well-established (18, 19) its involvement in the resolution of inflammation is an emerging area (10, 13, 20–24). We have shown previously that administration of Plg or Pla increases recruitment and migration of mononuclear cells (13), concomitantly with the induction of macrophage polarization toward anti-inflammatory and resolving phenotypes *in vivo* (24). It has been demonstrated that the key role that the Plg/Pla system plays in the phagocytosis of apoptotic cells requires interaction of Plg with the cell surface (21–24). However, specific plasminogen receptor(s) mediating this effect *in vivo* have not been identified. In the present study, we have investigated the effects of genetic deletion of either Plg, or its receptor Plg-R_KT_, on the resolution of inflammation. Deletion of either Plg or Plg-R_KT_ impaired recruitment of mononuclear cells to the pleural cavity of mice in a self-resolving model of pleurisy, concomitantly with an increased percentage of inflammatory M1-like macrophages. Furthermore, Plg/Pla treatment increased M2 markers, and decreased IFN/LPS-induced M1 markers *in vitro*. Accordingly, IL4-induced polarization of macrophages to an M2-like phenotype was impaired in the absence of Plg and Plg-R_KT_, and was associated with decreased phosphorylated STAT3. Moreover, deletion of either Plg or Plg-R_KT_ resulted in lower expression of pro-efferocytosis molecules *in vivo* resulting in lower engulfment of apoptotic neutrophils. Our results support a key role for Plg/Pla and Plg-R_KT_ in the resolution of the inflammatory response.

## Methods

### Animals

Male Plg-R_KT_ gene targeted mice (8–10 weeks of age) were backcrossed 10 generations into the C57Bl/6J background (17). Male mPlg-R_KT_^−/−^ (Plg-R_KT_ specifically deleted in myeloid cells) and Plg-R_KT_^flox/*flox*^ mice (8–10 weeks of age) were generated and characterized as described in Figure S1. Breeding pairs of Plg-deficient mice were a kind gift from Dr. Victoria Ploplis, University of Notre Dame, Indiana, USA. Male mice (8–10 weeks of age) were used for all experiments.

### Proteins

Human Plg was from Enzyme Research Laboratories, San Diego, CA and human Pla was from Sigma-Aldrich (St. Louis, MO, USA). We have demonstrated previously that mouse Pla and human Pla have similar activity in the LPS-induced pleurisy model (13).

### Leukocyte Migration Into the Pleural Cavity Induced by LPS

Mice received an intrapleural (i.pl.) injection of either LPS or PBS (vehicle) as described (24–26). Cells recruited to the cavity were recovered 24–48 h following injection by washing the cavity with 1 mL of PBS. Total cell counts were determined using Turk's stain in a modified Neubauer chamber. Differential cell counting was performed using standard morphological criteria to identify cell types on cyto-centrifuge preparations (Shandon Elliott) stained with May-Grünwald-Giemsa.

### Antibodies and Reagents

LPS from *Escherichia coli* (serotype O:111:B4) was from Sigma-Aldrich; IFN, IL-4, and IL-10 were from Biolegend (San Diego, CA, USA). Antibodies used for western blotting were anti-mouse arginase-1 and annexin A1 (Santa Cruz biotechnology, Dallas, TX, USA), p-STAT1, p-STAT3, and p-STAT6 (Cell Signaling Technology, Danvers, MA, USA) and anti-mouse β-actin and mouse/anti-rabbit secondary antibodies (LI-COR, Lincoln, NE, USA). Fluorescent monoclonal antibodies for flow cytometry were anti-mouse F4/80 (PE-Cy7 Invitrogen, Carlsbad, CA, USA), GR1 (APC-eBioscience/Thermo Fisher Scientific), CD11b (V500-BD Biosciences, Franklin Lakes, NJ, USA), CD206 (APC Biolegend), CD86 (PE, BD Biosciences), anti-rabbit secondary (alexa fluor 488-A11034 and alexa fluor 405-31556-Invitrogen); and anti-human HLA_DR/DP/DQ_ (FITC, BD Pharmingen) and anti-human CD86 (PE, Biolegend). ELISA kits for measurement of murine IL-10, TGF-β, CCL2, CXCL1, and TNF-α were from R&D Systems (Minneapolis, MN, USA); ELISA kits for measurement of human TNF-α and IL-10 were from eBioscience.

### Murine Bone Marrow-Derived Macrophages (BMDMs)

BMDMs were prepared as previously described (24) with modifications. Bone marrow was collected from tibias and femurs and washed with Dulbecco's Modified Eagles Medium (DMEM) containing penicillin 100 units/mL and streptomycin 100 μg/mL and the suspension obtained was then centrifuged for 5 min at 1,200 g. The pellet was resuspended with complete conditioned media for BMDM differentiation [DMEM with 10% heat-inactivated fetal bovine serum (FBS) and 20% L929 cell filtered supernatant media], seeded on tissue culture plates, and incubated at 37°C with 5% CO_2_. After 3 days, the medium was supplemented with additional complete conditioned media. At day 7 the supernatant was removed, and adherent macrophages were detached using a cell scraper and plated in 96-well-plates (Corning™ Costar™, Corning, NY, USA) (2 × 10^5^ cells/well) for flow cytometry or in 6-well plates (Corning™ Costar™) (2 × 10^6^ cells/well) for western blotting.

### Human Macrophages

Human macrophages were prepared as described (27). Briefly, whole blood was centrifuged at 130 × g for 20 min and plasma was removed. For every 10 mL of whole blood, erythrocytes were depleted by sequentially layering 8 mL of 6% w/v dextran (Sigma-Aldrich, Poole, UK) and 10 mL of PBS. After 15 min, the leukocyte-rich fraction was layered over Histopaque 1077 (Sigma-Aldrich) and centrifuged for 30 min at 450 × g at room temperature to separate granulocytes from peripheral blood mononuclear cells (PBMC). PBMC (20 × 10^6^ cells) were seeded in a petri dish. After 1 h incubation at 37 °C, cells were washed to remove lymphocytes and incubated with 50 ng/mL macrophage-colony stimulating factor (M-CSF, PeproTech, London, UK) in RPMI/FBS 10%. Medium was replaced on day 5. On day 7 macrophages were detached with cell detachment solution (Accutase™, Sigma-Aldrich) and seeded in 24-well plate (0.5 × 10^6^ cells/well) for flow cytometric analysis. Experiments using healthy volunteers were approved by the local research ethics committee (P/00/029 East London and The City Local Research Ethics Committee 1). Informed written consent was provided according to the Declaration of Helsinki.

### Flow Cytometric Analyses

Murine leukocytes were stained with fluorescent monoclonal antibodies against F4/80 (labeled with PE-Cy7,), GR1 (labeled with APC,), CD11b (labeled with V500), CD206 (labeled with APC), CD86 (labeled with PE), anti-rabbit secondary (labeled with alexa 488 and with alexa 405). Stained cells were acquired in a NovoCyte (ACEA Biosciences, San Diego, CA, USA) and analyzed using FlowJo software (Tree Star Inc., Ashland, OR, USA). Macrophage populations were defined according to F4/80, GR1, and CD11b expression, as previously described (24, 28). For human cell analyses, macrophages were labeled with 1.25 μg/mL anti-HLA_DR/DP/DQ_-FITC (BD Pharmingen) and 1 μg/mL anti-CD86-PE (BioLegend), at 4°C for 30 min. Cells were acquired on a LSRFortessa cytometer (BD Biosciences).

### qPCR Analysis of M1 and M2 Macrophage Markers

Total RNA from BMDMs was extracted using TRIzol Reagent (Invitrogen) according to the manufacturer's instructions. cDNA was synthetized using 500 ng of RNA with the SuperScript III Reverse Transcriptase (Invitrogen), according to the manufacturer's instructions. Real-time PCR was performed in duplicate, with obtained cDNA, specific primers and Power SYBR Green PCR Master Mix (Applied Biosystems, Foster City, CA, USA), using the StepOne™ System (Applied Biosystems). The data were analyzed using StepOne™ System software with a cycle threshold (Ct) in the linear range of amplification and then processed by the 2^−ΔΔ*Ct*^ method. The dissociation step was always included to confirm the absence of unspecific products. *Gapdh* was used as an endogenous control to normalize the variability in expression levels and results were expressed as fold increase. Primers used are listed in Supplemental Materials.

### Human Neutrophil Isolation and Efferocytosis Assay

Neutrophils were isolated from peripheral blood of healthy donors on a histopaque gradient (Histopaque 11191 and 10771, from Sigma-Aldrich) as previously described (24, 29). Neutrophil apoptosis was induced by treatment with 10 μM staurosporine (Sigma-Aldrich) for 1 h. Apoptotic neutrophils were labeled (for flow cytometric analysis) by incubation with 5 μM CFSE (carboxyfluorescein diacetate succinimidyl ester—Life Technologies, Carlsbad, CA, USA) at 37°C and 5% CO_2_ for 1 h. The percentage of apoptosis was determined in cytospin preparations, counted using oil immersion microscopy (100x objective) to determine the proportion of cells with high distinctive apoptotic morphology (25, 29, 30) and >90% were apoptotic. Apoptosis induction by staurosporine was also verified by flow cytometry using Annexin V-FITC and propidium iodide (BD Pharmingen).

The *in vivo* efferocytosis assay was performed as previously described (24, 31, 32). Mice received an intraperitoneal (i.p.) injection of zymosan (0.1 mg/mouse) to induce peritonitis. After 72 h, mice were injected i.p. with 3 × 10^6^ apoptotic human neutrophils labeled with CFSE. One hour later, mice were euthanized and the cells in the peritoneal cavity were recovered by washing the cavity with 5 mL of PBS.

The *in vitro* efferocytosis assay was performed by co-culturing BMDMs with human or mouse apoptotic neutrophils labeled with CFSE in a proportion of 3 apoptotic neutrophils: 1 macrophage. Mouse neutrophils were obtained from bone marrow and isolated on a using histopaque gradient (Histopaque 11191 and 10771 from Sigma-Aldrich) as described previously (33). Neutrophils that had not been phagocytosed were removed 1 h later by vigorous washing of the wells with PBS 3 times. Efferocytosis by adherent macrophages was assessed by flow cytometric analyses using the frequency of F480^+^/CFSE^+^ cells, and the results were expressed as mean florescence intensity (MFI) of CFSE (flow cytometer laser set at 488). Efferocytosis was also assessed on cytospin preparations of cells after staining with May-Grunwald-Giemsa by determining the proportion of macrophages that ingested apoptotic neutrophils (500 cells/slides were counted). Results are presented as efferocytosis index.

### Western Blotting

Cells were lysed in RIPA buffer containing anti-protease and anti-phosphatase cocktail (Thermo Fisher Scientific). Cell lysates were electrophoresed on denaturing 4–15% polyacrylamide-SDS gels under reducing conditions and electrotransferred to nitrocellulose membranes. Membranes were incubated with primary antibodies, washed with PBS-Tween-20 0.1% and incubated with species specific IRDye®680RD/800CW-conjugated secondary antibodies. Immunoreactive bands were visualized using the Odyssey Imaging System (LI-COR), according to the manufacturer's instructions (Biosciences). For densitometric analysis, membranes were scanned and quantified using the software Image Studio™ Lite Software 5.2 (LI-COR).

### Statistical Analysis

Data are as mean ± SEM. The statistical significance between groups was determined by Student-Newman-Keuls *post-hoc* test, unless otherwise indicated. A *P* < 0.05 was considered significant. Calculations were performed using the prism 5.0 software program for Windows (GraphPad software, San Diego, CA, USA).

### Study Approval

All animal experiments were approved by the Institutional Animal Care and Use Committee of The Scripps Research Institute.

## Results

### Genetic Deletion of Either Plg or Plg-R_KT_ Results in Decreased Mononuclear Cell Recruitment and Decreased Levels of Pleural CCL2 in a Self-Resolving Model of Pleurisy

**Figure 1:**
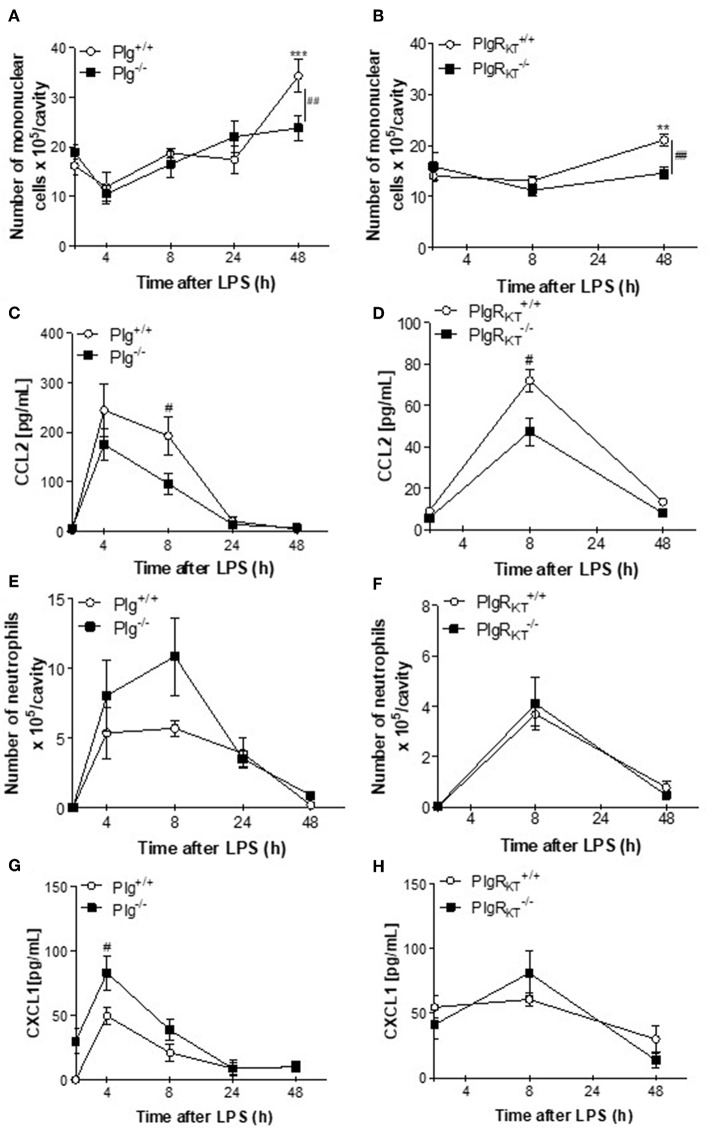
Time-course of leukocyte recruitment during LPS-induced pleurisy in Plg^−/−^ and Plg-R_KT_^−/−^ mice and their respective wild type littermates. Plg^+/+^, Plg^−/−^, Plg-R_KT_^+/+^, and Plg-R_KT_^−/−^ mice were injected intrapleurally with LPS (250 ng/cavity, i.pl.) or PBS. Cells present in the pleural cavity were harvested 4, 8, 24, and 48 h after LPS challenge. The number of mononuclear cells **(A,B)** and neutrophils **(E,F)** were evaluated by counting cytospin slides stained with May-Grunwald-Giemsa. The levels of the monocyte chemoattractant CCL2 **(C,D)** and the neutrophil chemoattractant, CXCL1 **(G,H)** were quantified in cell-free pleural lavages by ELISA. Results are shown as the mean ± SEM of at least five mice per group. **P < 0.01 and ***P < 0.001, when comparing LPS-injected mice with PBS-injected mice. ^#^*P* < 0.05 and ^##^*P* < 0.01, when comparing wild type and knockout mice injected with LPS.

To examine the impact of Plg and Plg-R_KT_ deficiency on the resolution of inflammation, we utilized a well-established model of LPS-induced inflammation. In this model, intrapleural injection of LPS induces a time-dependent influx of leukocytes into the pleural cavity, characterized by early neutrophilic infiltration, with resolution at 48 h, when neutrophils are scarce and the number of mononuclear cells is maximal (25, 26). Plg^−/−^ and Plg-R_KT_^−/−^ mice and their respective wild type (WT) littermate controls (Plg^+/+^ and Plg-R_K_^+/+^ mice) were challenged with LPS or PBS and pleural cavities were washed 4, 8, 24, and 48 h later. In Plg^+/+^ and Plg-R_K_^+/+^ mice, the number of mononuclear cells recruited to the pleural cavity in response to LPS injection increased with time (Figures 1A,B). At 48 h, the number of mononuclear cells recruited to the pleural cavity was significantly lower in Plg^−/−^ and Plg-R_KT_^−/−^ mice than their respective WT controls (Figures 1A,B). Pleural levels of the chemokine CCL2 [a chemoattractant for monocytes and lymphocytes (34)] were significantly decreased at 8 h in Plg^−/−^ and Plg-R_KT_^−/−^ mice (Figures 1C,D), consistent with the decreased mononuclear cell recruitment in these mice. There was no significant effect of either genotype on neutrophil recruitment (Figures 1E,F), although there was a trend for increased neutrophil recruitment in Plg^−/−^ mice (Figure 1E), consistent with increased levels of the neutrophil chemoattractant, CXCL1 (Figure 1G). However, and in accordance with the similar profile of neutrophil recruitment in Plg-R_KT_^+/+^ and Plg-R_KT_^−/−^ mice, no difference in CXCL1 levels between these two genotypes was detected (Figure 1H). Interestingly, mPlg-R_KT_^−/−^ mice in which Plg-R_KT_ was specifically deleted in myeloid cells (Figure S1), did not differ in the number of mononuclear cells recruited into the pleural cavity or in levels of CCL2 compared with Plg-R_KT_^*flox*/*flox*^ controls (Figures S2A,B). Thus, Plg-R_KT_ deficiency in other cell types in addition to myeloid cells is likely to impact Plg-R_KT_-dependent monocyte recruitment. There was also no effect of specific deletion of Plg-R_KT_ in myeloid cells on the number of recruited neutrophils or pleural levels of CXCL1 (Figures S2C,D), consistent with the results obtained with mice with global Plg-R_KT_ deletion.

### Genetic Deletion of Either Plg or Plg-R_KT_ Results in an Increased Proportion of Proinflammatory Macrophages in a Self-Resolving Model of Pleurisy

**Figure 2:**
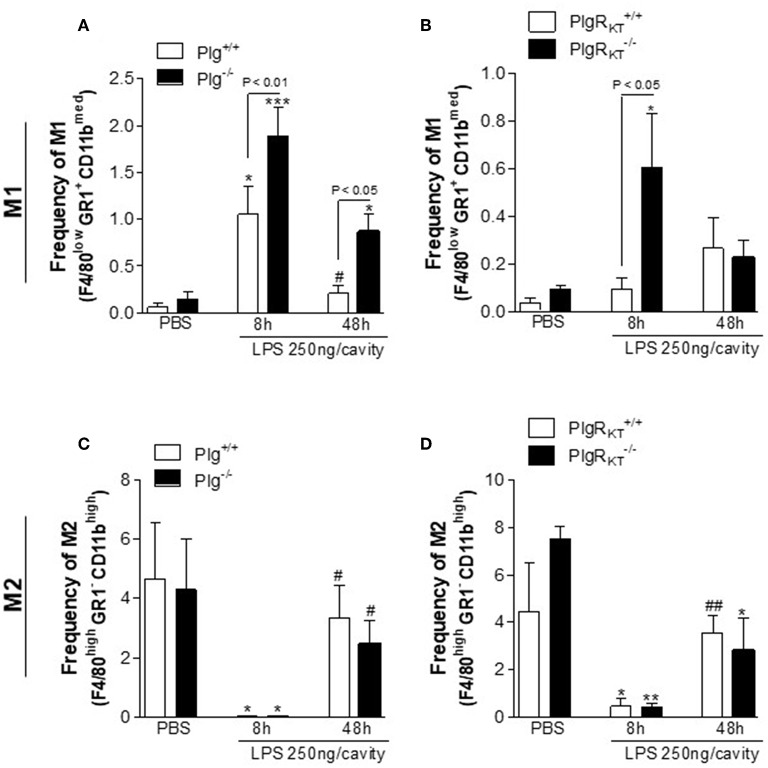
Characterization of macrophage polarization in Plg^−/−^ and Plg-R_KT_^−/−^ mice and their respective wild type littermates during LPS-induced pleurisy. Plg^+/+^, Plg^−/−^, Plg-R_KT_^+/+^, and Plg-R_KT_^−/−^ mice were injected with either LPS (250 ng/cavity, i.pl.) or PBS. Cells present in the pleural cavity were harvested at 8 and 48 h after LPS challenge or PBS injection. The percentage of M1-like [F4/80^low^ GR1^+^ CD11b^med^] **(A,B)** and M2-like [F4/80^high^ GR1^−^ CD11b^high^] **(C,D)** macrophage populations were determined by flow cytometry. Results are shown as the mean ± SEM of at least four mice in each group. *P < 0.05, **P < 0.01, and ***P < 0.001, when comparing 8 h LPS-injected with PBS-injected mice; ^#^*P* < 0.05 and ^##^*P* < 0.01, when comparing cells harvested 48 h after LPS injection with cells harvested with 8 h after LPS injection mice.

In the LPS-induced pleurisy model, proinflammatory macrophages are the predominant macrophage phenotype present in the pleural space 8 h after LPS injection (the productive phase of inflammation). Anti-inflammatory macrophages are the predominant macrophage phenotype present in the resident pleural macrophage population (PBS-injected) and are also predominant at 48 h following LPS treatment (the resolving phase) (28). Interestingly, Plg expression as well as Pla activity are increased in the resolving phase of this model (24). Therefore, we analyzed whether deletion of either Plg or Plg-R_KT_ would affect the proportion of macrophage phenotype at different time points. In flow cytometric analyses, we found increased proportion of proinflammatory M1 macrophages (F4/80^low^ GR1^+^ CD11b^med^) in the pleural cavities of Plg^−/−^ and Plg-R_KT_^−/−^ mice during the active phase of inflammation (8 h) compared with their respective WT controls (Figures 2A,B). There were no significant genotype-dependent effect on the proportion of M2-like macrophage (F4/80^high^ GR1^−^ CD11b^high^) (Figures 2C,D), suggesting that deletion of either Plg or Plg-R_KT_ causes impairment in polarization to the anti-inflammatory (M2) phenotype and thus, macrophages retain the M1-like phenotype.

### Treatment of BMDMs With Either Plg or Pla Increases Expression of M2 Markers and M2 Secretory Products, While Decreasing M1 Marker Expression

**Figure 3:**
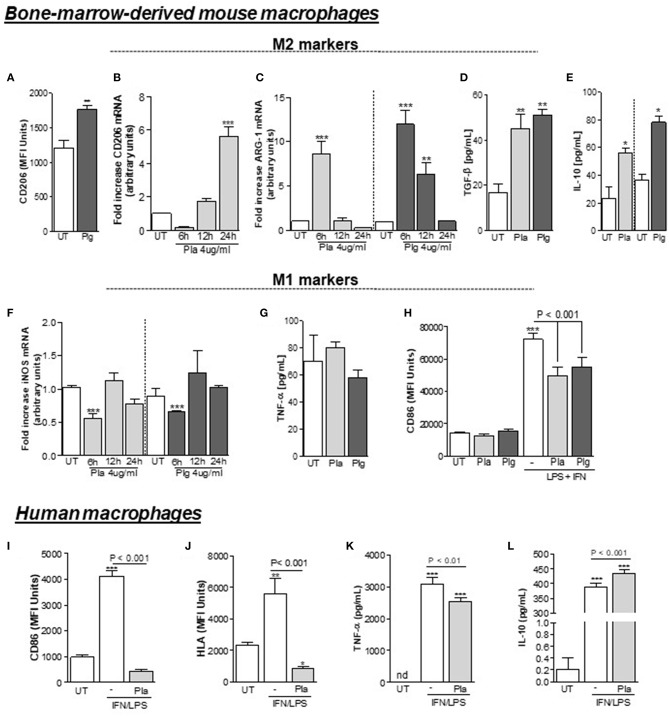
Effect of Plg and Pla on macrophage polarization. BMDMs from C57BL/6J WT mice were washed 3 times with serum free DMEM, treated with Plg (4 μg/mL) or Pla (4 μg/mL) or untreated (UT) for the indicated times and analyzed by flow cytometry **(A)** or qPCR **(B)** for expression of the M2 marker CD206, and by qPCR for expression of the M2 marker, arginase-1 **(C)**. Levels of the secretory products of M2-like macrophages, TGF-β **(D)** and IL-10 **(E)** were quantified 24 h after treatments in conditioned media by ELISA. Treated and untreated BMDMs were also analyzed for expression of the M1 marker iNOS by qPCR **(F)** and conditioned media collected at 24 h were analyzed by ELISA for concentrations of the secretory product of M1-like macrophages, TNF-α **(G)**. BMDMs **(H)** and human macrophages **(I–L)** were pre-treated with Plg or Pla (both at 4 μg/mL) for 1 h, and then, stimulated with LPS (10 ng/mL) + IFN (10 ng/mL) and with LPS (10 ng/mL) + IFN (20 ng/mL) for 24 h, respectively. The expression of the M1 markers CD86 **(H,I)** and HLA **(J)** was measured using flow cytometry and conditioned media were subjected to ELISA for detection of human TNF-α **(K)** or IL-10 **(L)**. nd, not-detected. *P < 0.05, **P < 0.01, and ***P < 0.001 when comparing Plg- or Pla-treated with untreated (UT) BMDMs or untreated human macrophages.

Based on the results above, we investigated the effect of either Plg or Pla treatment on macrophage polarization *in vitro*, using WT C57Bl/6J BMDMs. Significantly increased expression of the M2 markers CD206 (Figures 3A,B) and arginase-1 (Figure 3C) was observed following cell incubation with Plg/Pla. Also, in response to Plg/Pla treatment, conditioned media harvested from treated BMDMs had significantly increased levels of cytokines typically secreted by M2-like macrophages, TGF-β (Figure 3D) and IL-10 (Figure 3E). In contrast, Plg/Pla downregulated BMDM expression of the M1 marker, iNOS (Figure 3F). No effect of Plg/Pla treatment was observed on secretion of the proinflammatory cytokine TNF-α (Figure 3G). Interestingly, pretreatment of BMDMs with Plg/Pla reduced IFN/LPS-induced upregulation of the M1 marker, CD86 (Figure 3H). In parallel experiments with human macrophages, pretreatment with Pla inhibited IFN/LPS-induced expression of the M1 markers, CD86 and HLA (Figures 3I,J) and secretion of the M1-like product, TNF-α (Figure 3K), while increasing secretion of the M2-like secretory product, IL-10 (Figure 3L). Taken together these results suggest that Plg and Pla promote macrophage polarization to the M2-like phenotype while decreasing expression of IFN/LPS-induced M1 phenotypic markers in both mouse and human macrophages.

### IL4-Induced Macrophage Polarization Toward an M2-Like Phenotype Is Impaired in Plg^−/−^ and Plg-R_KT_^−/−^ BMDMs

**Figure 4:**
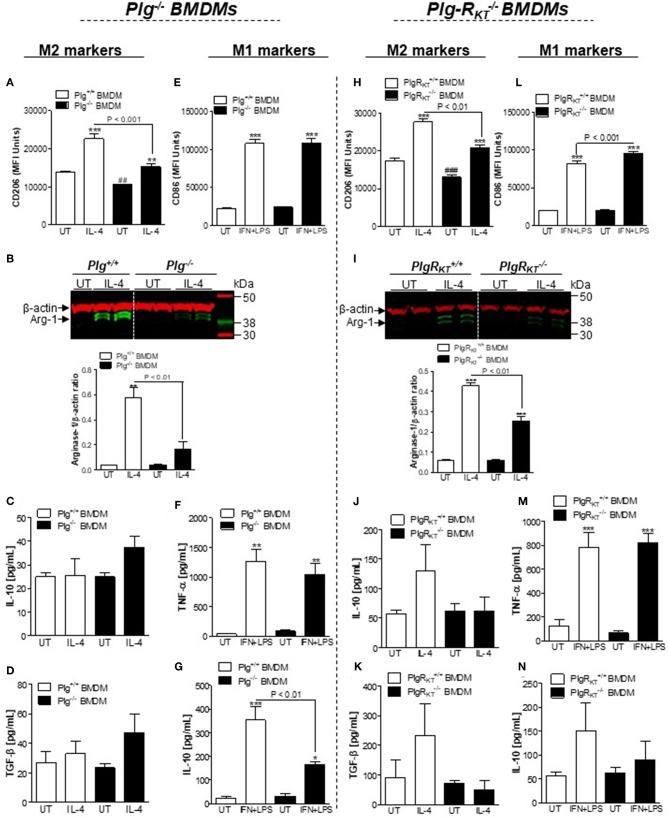
Effects of deletion of either Plg or Plg-R_KT_ on macrophage polarization. BMDMs from Plg^+/+^, Plg^−/−^, Plg-R_KT_^+/+^, and Plg-R_KT_^−/−^ mice were separately washed 3 times with serum free media DMEM and then were either untreated (UT) or stimulated with IL-4 (20 ng/mL) or LPS (10 ng/mL) + IFN (10 ng/mL) for 24 h. Cells were analyzed by flow cytometry for expression of the M2 markers CD206 **(A,H)** and the M1 marker CD86 **(E,L)**. Expression of the M2 marker, arginase-1 **(B,I)**, was determined by Western blotting with anti-β-actin used as loading control. The levels of secretory products of M2-like macrophages TGF-β **(D,K)** and IL-10 **(C,J,G,N)**, and M1-like macrophages TNF-α **(F,M)** were determined in conditioned media by ELISA. *P < 0.05, **P < 0.01, and ***P < 0.001 when comparing treated with untreated (UT) BMDMs and ^##^*P* < 0.001 and ^###^*P* < 0.001 when comparing UT wild type with UT knockout BMDMs.

We next examined the response of BMDMs from Plg^−/−^ and Plg-R_KT_^−/−^ mice to standard macrophage polarizing agents to induce either the M1-like (IFN+LPS) or the M2-like phenotype (IL-4). Expression of CD206 and arginase-1 following stimulation with IL-4 was lower in Plg^−/−^ and Plg-R_KT_^−/−^ BMDMs compared with their WT controls (Figures 4A,B,H,I, respectively), consistent with a requirement for both Plg and its receptor, Plg-R_KT_, in macrophage polarization to the M2-like phenotype. In contrast, in these settings TGF-β and IL-10 levels were not affected by deletion of either Plg or Plg-R_KT_ (Figures 4C,D,J,K, respectively). No impairment of expression of markers of polarization to the M1-like phenotype CD86 (Figure 4E) and TNF-α (Figure 4F) was observed in Plg^−/−^ BMDMs following treatment with IFN/LPS. Interestingly, we found an increased expression of CD86 without modification in TNF-α levels after IFN+LPS stimulation of Plg-R_KT_^−/−^ BMDMs (Figures 4L,M). Levels of IL-10 were reduced in IFN/LPS-stimulated Plg^−/−^ BMDMs (Figure 4G) with a trend for reduction in IL-10 levels in Plg-R_KT_^−/−^ BMDMs (Figure 4N).

We also examined the responses to either IFN+LPS or to IL-4 by BMDMs from mPlg-R_KT_^−/−^ mice. Expression of M1-like and M2-like markers by mPlg-R_KT_^−/−^ BMDMs mirrored that observed with Plg-R_KT_^−/−^ BMDMs. In response to IL-4 treatment, expression of CD206 was impaired (Figure S3A), while expression of the M2 secretory products IL-10 and TGF-β, were not significantly different than with Plg-R_KT_^*flox*/*flox*^ controls (Figures S3B,C). Results with LPS+IFN stimulation also were in parallel with those of Plg-R_KT_^−/−^ BMDMs with an increase in expression of CD86 (Figure S3D) without modification in IL-10 levels (Figure S3F). In contrast, TNF-α levels were increased in conditioned media of mPlg-R_KT_^−/−^ BMDMs (Figure S3E). Taken together, these results suggest that deficiency of either Plg or Plg-R_KT_ impairs macrophage polarization toward an M2-like phenotype.

### Deletion of Either Plg or Plg-R_KT_ Results in Reduced STAT3 Phosphorylation in Macrophages

**Figure 5:**
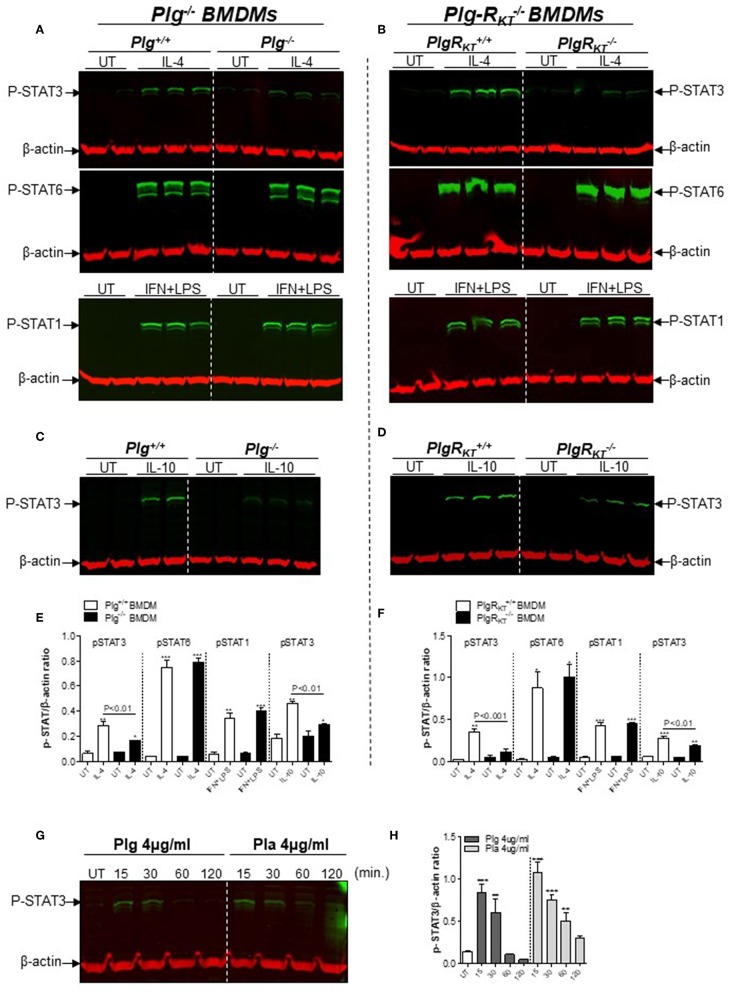
Effect of plasmin(ogen) treatment and deletion of either Plg or Plg-R_KT_ on STAT signaling pathways. BMDMs from Plg^+/+^, Plg^−/−^, Plg-R_KT_^+/+^, and Plg-R_KT_^−/−^ mice were washed 3 times with serum free media and then treated with either IL-4 (20 ng/mL), LPS (10 ng/mL) + IFN (10 ng/mL), IL-10 (20 ng/mL), or untreated (UT) for 30 min **(A–D)**. BMDMs from wild type C57Bl/6J mice were washed 3 times with serum free media and then treated with Plg or Pla (each at 4 μg/mL) for 15–120 min or untreated (UT) for 30 min **(G,H)**. Cell lysates were analyzed by Western blotting for the indicated antigens. β-actin was used as loading control. β-actin was used as a loading control. Densitometry analyses are shown **(E,F,H)**. *P < 0.05, **P < 0.01, and ***P < 0.001 when comparing treated with UT BMDMs.

Standard macrophage polarizing agents such as IL-4 induce an M2-like phenotype through both STAT6 (canonical pathway) and STAT3 (non-canonical pathway), and IFN+LPS induces macrophage polarization toward the M1-like phenotype via STAT1 (35). Therefore, we investigated the effect of deletion of either Plg or Plg-R_KT_ on levels of pSTAT3, pSTAT6, or pSTAT1 during polarization of BMDMs. Levels of pSTAT3 were increased following IL-4 treatment in Plg^+/+^ and Plg-R_KT_^+/+^ BMDMs, while the response was markedly and significantly decreased in Plg^−/−^ and Plg-R_KT_^−/−^ BMDMs (Figures 5A,B,E,F). There was no genotype effect on IL4-induced levels of pSTAT6 or IFN+LPS-induced levels of pSTAT1 (Figures 5A,B,E,F). Results with mPlg-R_KT_^−/−^ BMDMs mirrored those with Plg-R_KT_^−/−^ BMDMs (Figure S4A).

Because STAT3 is a component of the canonical pathway induced during M2 polarization in response to IL-10 treatment (36), we measured levels of pSTAT3 following IL-10 treatment of BMDMs. pSTAT3 levels were markedly lower in BMDMs from Plg^−/−^, Plg-R_KT_^−/−^ and mPlg-R_KT_^−/−^ mice compared with BMDMs from control littermates (Figures 5C,D and Figure S4B). In a complementary manner, we measured secreted TGF-β as an additional readout of IL10-induced M2-like polarization and found lower levels in conditioned media from Plg^−/−^ and mPlg-R_KT_^−/−^ BMDMs compared to their respective WT controls (Figures S5A,B). Since phosphorylated-STAT3 levels were lower in Plg^−/−^ and Plg-R_KT_^−/−^ BMDMs (Figures 5A–F), we tested whether treatment with either Plg or Pla would affect pSTAT3. Treatment of WT C57Bl/6J BMDMs with either Plg or Pla resulted in transient increases in levels of pSTAT3 within 15–30 min, decreasing at 60–120 min (Figures 5G,H). Taken together these results suggest that Plg and Plg-R_KT_ mediate polarization of macrophages to the M2-like phenotype via STAT3 signaling.

### Deletion of Either Plg or Plg-R_KT_ Results in Decreased Expression of Engulfment Molecules *in vivo*

**Figure 6:**
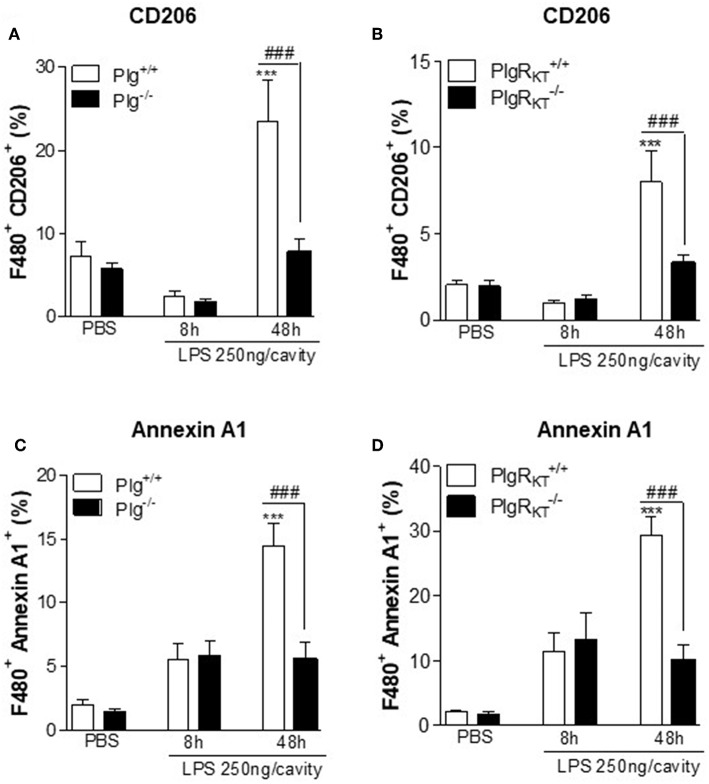
Expression of engulfment molecules during LPS-induced pleurisy. Plg^+/+^, Plg^−/−^, Plg-R_KT_^+/+^, and Plg-R_KT_^−/−^ mice were injected with LPS (250 ng/cavity, i.pl.) or PBS. Cells present in the pleural cavity were harvested 8 and 48 h after LPS challenge or after PBS injection (48 h). The frequency of F4/80^+^ CD206^+^ **(A,B)** and F4/80^+^ Annexin A1^+^ **(C,D)** double positives cells was determined by flow cytometry. Results are shown as the mean ± SEM of at least four mice in each group. ***P < 0.001, when comparing LPS-injected mice with PBS-injected mice and ^###^*P* < 0.01 when comparing LPS-injected wild type with knockout mice.

M2 macrophages play a key role in efferocytosis (6, 7). Therefore, we examined the impact of Plg or Plg-R_KT_ deficiency on expression by macrophages of representative molecules known to regulate efferocytosis of apoptotic cells, CD206 and Annexin A1 (AnxA1) (37, 38). During the resolving phase of LPS-induced pleurisy (48 h), the frequency of cells doubly positive for F4/80/CD206 (Figures 6A,B) and doubly positive for F4/80/AnxA1 (Figures 6C,D) was markedly decreased in the pleural exudates of both Plg^−/−^ and Plg-R_KT_^−/−^ mice compared to their respective WT littermate controls. These results suggest that Plg and Plg-R_KT_ contribute to the expression of molecules associated with M2-like macrophages and with efferocytosis during the resolution phase of inflammation.

### Deletion of Either Plg or Plg-R_KT_ Results in Decreased Efferocytosis of Apoptotic Neutrophils *in vivo*

**Figure 7:**
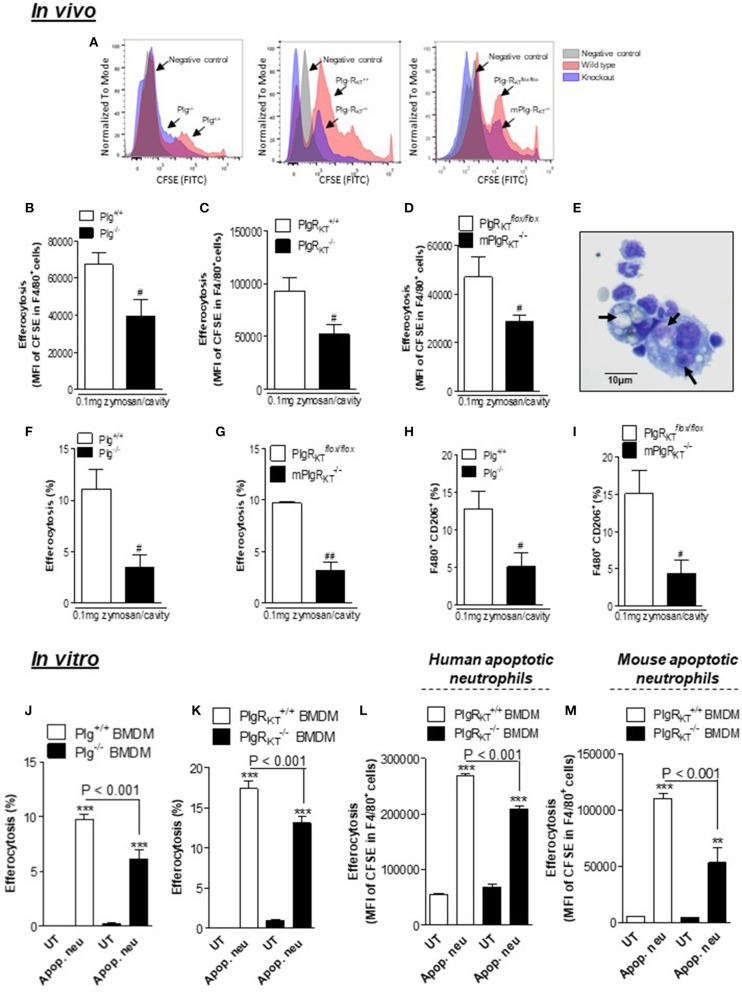
Effect of deletion of Plg or its receptor, Plg-R_KT_, on efferocytosis. To determine efferocytosis *in vivo*, Plg^+/+^, Plg^−/−^, Plg-R_KT_^+/+^, Plg-R_KT_^−/−^, mPlg-R_KT_^−/−^, and Plg-R_KT_^-flox/*flox*^ mice received an i.p. injection of 0.1 mg of zymosan and 71 h later received an i.p. injection of 3 × 10^6^ apoptotic human neutrophils labeled with fluorescent CFSE. The cells from the peritoneal cavity were collected 1 h later. The *in vitro* efferocytosis assay was performed by co-culturing BMDMs with either human or mouse apoptotic neutrophils labeled with CFSE in a proportion of 3 neutrophils per macrophage. Efferocytosis was assessed by flow cytometry analyzing MFI (mean fluorescence intensity) of F4/80^+^ FITC^+^ **(B–D,L,M)** and by counting cytospin slides **(F,G,J,K)**. Representative histograms **(A)** and images of neutrophils inside macrophages **(E)** are shown (arrows). Magnification 40X. The frequency of F4/80^+^ CD206^+^ **(H,I)** double positives cells were determined by flow cytometry. Flow cytometry data are expressed as MFI or frequency, and are shown as the mean ± SEM of at least four mice in each group. **P < 0.01 and ***P < 0.001, when comparing BMDMs treated with apoptotic neutrophils with untreated (UT) BMDMs and ^#^*P* < 0.05 and ^##^*P* < 0.01 when comparing zymosan-injected knockout with control mice.

Next, by using an *in vivo* model of efferocytosis (24, 31, 32) we tested whether deletion of either Plg or Plg-R_KT_ would affect the ability of macrophages to phagocytose apoptotic neutrophils (efferocytosis). Mice received an intraperitoneal injection of zymosan (0.1 mg/cavity) to induce macrophage recruitment and were then injected intraperitoneally with human apoptotic neutrophils labeled with fluorescent CFSE as described (24, 31, 32). Cells were collected from the peritoneal exudate 1 h later for flow cytometry. Representative histograms are shown in Figure 7A. Engulfment of apoptotic neutrophils was lower in Plg^−/−^, Plg-R_KT_^−/−^, and mPlg-R_KT_^−/−^ mice compared to their littermate controls, as seen by flow cytometry (Figures 7B–D) or by counting the percentage of macrophages that had ingested apoptotic neutrophils, on cytospin slides (Figures 7F,G). Representative images are shown in Figure 7E. Interestingly, the frequency of macrophages expressing CD206 during efferocytosis was also significantly decreased in Plg^−/−^ and mPlg-R_KT_^−/−^ mice relative to their WT controls (Figures 7H,I).

We also examined efferocytosis *in vitro*. By counting cytospin slides we found that % efferocytosis by both Plg^−/−^ and Plg-R_KT_^−/−^ BMDMs was decreased relative to BMDMs from their respective WT controls (Figures 7J,K). By FACS, we observed similar effects with Plg-R_KT_^−/−^ BMDMs when using either human or mouse neutrophils as prey (Figures 7L,M). These results indicate that Plg and Plg-R_KT_ function to regulate macrophage polarization and efferocytosis of apoptotic cells both *in vivo* and *in vitro*.

## Discussion

The role of the Plg/Pla system in the resolving phase of inflammation and the receptor-dependence of key steps in the resolution of inflammation is an emerging area (24). In the current study, utilizing both Plg and Plg-R_KT_-deficient mice, we found that: (1) Optimal mononuclear cell recruitment in response to LPS-induced pleurisy required both Plg and the Plg receptor, Plg-R_KT_; (2) Optimal polarization of macrophages to the anti-inflammatory M2-like phenotype required both Plg/Pla and Plg-R_KT_; (3) Stimulation of key intracellular signaling events for macrophage polarization required both Plg/Pla and Plg-R_KT_; (4) Expression of engulfment molecules and effective efferocytosis required both Plg/Pla and Plg-R_KT_. Therefore, we provide evidence that endogenous Plg and Plg-R_KT_ regulate key steps in the resolution of inflammation by regulating monocyte/macrophage migration, reprogramming macrophages toward an M2-like phenotype, regulating cytokine release and promoting efferocytosis.

Monocyte migration leading to macrophage infiltration at inflammatory sites is a hallmark of the resolution of inflammation (39, 40). These cells promote clearance of the neutrophilic infiltrate, to prevent apoptotic neutrophils from becoming necrotic and pro-inflammatory (5). Plg/Pla is required for optimal macrophage recruitment in response to a diverse array of inflammatory stimuli (10, 13, 41, 42). This requires association of Plg/Pla with C-terminal lysines exposed on the surfaces of monocytes/macrophages (11, 13, 16, 43, 44) and the proteolytic activity of Pla (10, 13). We have shown that Pla-induced macrophage chemotaxis *in vitro* and mononuclear cell recruitment *in vivo* are dependent on lysine binding sites and Pla activity (13). Here, utilizing a self-resolving model of LPS-induced pleurisy, we observed impaired recruitment of mononuclear cells in both Plg^−/−^ and Plg-R_KT_^−/−^ mice, consistent with previous studies in a model of sterile peritonitis (10, 16, 41). Moreover, our results provide the first evidence for decreased levels of the macrophage chemoattractant, CCL2, in Plg-R_KT_^−/−^ mice after inflammatory stimulation, consistent with the impaired mononuclear cell recruitment. Surprisingly, mononuclear cell recruitment and peritoneal CCL2 levels were not impaired in mice with Plg-R_KT_ deleted specifically in myeloid cells, suggesting that Plg-R_KT_ deficiency in other cell types such as mesothelial cells (45) may impact the production and/or processing of CCL2 and contribute to decreased monocyte migration in mice with globally deleted Plg-R_KT._ CCL2 can be produced by several cell types including endothelial, mesothelial, fibroblasts, epithelial, smooth muscle, mesangial, astrocytic, monocyte/macrophage, and microglial cells (45–49). Cailhier et al. have suggested that mesothelial cells are responsible for the production of this chemokine in the model of carrageenan-induced pleurisy (50). The results in the carrageenan-induced pleurisy model may be applicable to LPS-induced pleurisy as well. Pla has been shown to stimulate cytokine release by several cell types (19) and this may be dependent on plasminogen binding to Plg-R_KT_.

In Plg^−/−^ mice impaired macrophage migration also was associated with reduced pleural levels of CCL2 in LPS-induced pleurisy. A previous study found decreased peritoneal levels of CCL2 (~50% lower), associated with decreased macrophage recruitment after biomaterial implantation in Plg^−/−^ mice (42). We have previously shown that Plg/Pla treatment increases CCL2 levels and promotes monocyte migration to the pleural cavity of mice in a manner dependent on the CCL2/CCR2 axis (13), an effect also reported *in vitro* with human monocytes (51). Previous studies showed that Pla increases CCL2 chemotactic potency by cleavage at lysine 104 (52), and Pla may be indispensable to full activation of this chemokine *in vivo* (53). However, Plg-R_KT_-bound Pla proteolytic activity is likely necessary, but not sufficient for optimal recruitment of mononuclear cells, because mPlg-R_KT_^−/−^ mice are expected to have impaired surface expression of Pla, yet there is no effect on mononuclear cell recruitment in this model in mPlg-R_KT_^−/−^ mice. Although Plg^−/−^ and Plg-R_KT_^−/−^ mice exhibited increased polarization to M1-like macrophages following LPS challenge they did not exhibit prolonged inflammation, as determined by the presence of similar numbers of pleural neutrophil over time, compared to their wild-type littermate controls. This is consistent with previous studies in which Plg^−/−^ mice exhibited reduced mononuclear cell recruitment into the peritoneal cavity of thioglycolate-challenged mice, while the extent of neutrophil recruitment was similar to that of wild-type mice (10, 41) and with our previous study in which injection of Pla into the pleural cavity selectively induced the recruitment of mononuclear cells, but not neutrophils (13, 24). With regard to effects on other neutrophil functions, previous studies have demonstrated that, in contrast to monocytes, macrophages and dendritic cells, Pla does not directly activate neutrophils (19).

Coexistence of macrophages in different activation states and mixed phenotypes is observed at inflammatory sites. The distinct functional phenotypes can be induced *in vivo* or *in vitro* by inducers either alone, or in combination, including IFN, immune complexes, helminth infections, LPS, complement components, apoptotic cells, macrophage colony stimulating factor (M-CSF), glucocorticoids, IL-4, IL-13, IL-10, and TGF-beta (35, 36). Exposure to different activators results in a spectrum of macrophages phenotypes distinguished by variations in functions and expression/secretion of distinct molecules. For example, macrophages can differentiate to M1-like or a spectrum of M2-like subtypes (M2a, M2b, M2c, and M2d) depending on the applied stimuli (54). During the resolving phase of LPS-induced inflammation, macrophages are reprogramed toward resolving phenotypes (28). Here, in the model of LPS-induced pleurisy, we observed a greater percentage of M1-like macrophages in pleural exudates of both Plg^−/−^ and Plg-R_KT_^−/−^ mice, compared with their respective WT littermate controls. Interestingly, treatment with Plg/Pla reduced the expression of CD86 by M1-like BMDMs stimulated with IFN+LPS and decreased the expression of CD86 and HLA in M1-like human macrophages. Similarly, Borg et al. found decreased expression of CD86 and HLA by monocyte-derived dendritic cells in response to Pla treatment (23). Correspondingly, we found increased expression of CD86 in M1-like BMDMs from Plg-R_KT_-deficient mice. Also, Plg/Pla treatment decreased iNOS expression in BMDMs as previously observed *in vivo* in cells harvested from the pleural cavity (24). In addition, Plg/Pla treatment did not affect TNF-α levels, whereas Pla decreased TNF-α secretion and increased IL-10 secretion in human M1-like cells, suggesting a broad spectrum of Pla action to downregulate markers of pro-inflammatory macrophages. Moreover, Plg/Pla treatment increased expression of the M2 markers, Arginase-1/CD206, and secretion of IL-10 and TGF-β by BMDMs, similar to the effect of Plg/Pla treatment on TGF-β secretion by dendritic cells *in vitro* (23) and similar to the effect of intrapleural injection of Plg/Pla *in vivo* (24). Akin to these findings, polarization of BMDMs from Plg^−/−^ and Plg-R_KT_^−/−^ toward an M2-like phenotype with either IL-4 or IL-10 was impaired. Previous studies have shown that urokinase-type plasminogen activator (uPA) induces polarization of macrophages to an M2-like phenotype and prevents the M0 to M1 transition (55). In addition, uPA receptor (uPAR) deficient mice show increased expression of M1 markers and decreased M2 markers in gut tissue in a model of colitis (56). Taken together with our current data, effects of Pla on macrophage polarization are likely to require both proteolysis and receptor-dependent signaling.

The signal transduction and activators of transcription (STATs) convey the signaling of many inducers of the M2-like phenotype, including the cytokines IL-4, IL-10, and IL-6 (57–59), and the proresolving molecules AnxA1 (60) and maresins (61). Pla induces rapid phosphorylation of STAT3 in monocytes (51). And, Pla triggers activation and nuclear translocation of STAT3, but not of STAT1 or STAT5 in macrophages (62). In a murine model of sepsis, pSTAT3 expression is reduced in Plg^−/−^ mice compared to wild type control mice (63). IL-4 induces macrophage polarization toward the M2-like phenotype through both STAT6 (canonical pathway) and STAT3 (non-canonical pathway), and IFN/LPS treatment induces macrophage polarization toward the M1-like phenotype via STAT1 (35). Here we found decreased levels of pSTAT3, but not of pSTAT6, following treatment of either Plg^−/−^ or Plg-R_KT_^−/−^ BMDMs with inducers of M2 polarization (IL-4 or IL-10) compared to WT BMDMs. Mechanistically, our results suggest that Plg/Pla and Plg-R_KT_ mediate macrophage polarization to the M2 phenotype via STAT3 signaling. We suggest that Plg/Pla interacts with Plg-R_KT_ to mediate such polarization. This is the first report of a role for Plg-R_KT_ in intracellular signaling. As only a 4 amino acid sequence of Plg-R_KT_ has a cytoplasmic location, we expect that signaling will require interaction with other adjacent molecules that then become phosphorylated, as in the case of uPAR that mediates intracellular signaling despite being a GPI-linked receptor (64).

Engulfment of apoptotic cells is crucial for the resolution of inflammation and resumption of tissue homeostasis and prevents chronic inflammation and autoimmunity (5). The process of efferocytosis is mediated predominantly by M2-like macrophages (6, 65). Plg promotes clearance of apoptotic cells and this requires Plg activation, Pla activity, interaction of the lysine binding sites of plasmin(ogen) with C-terminal lysines expressed on the cell surface as well as *de novo* protein synthesis (21–24). Our data implicate, a specific receptor, Plg-R_KT_ (a plasminogen receptor exposing a C-terminal lysine on the cell surface) as a regulator of plasmin-mediated efferocytosis. Impaired efferocytosis in Plg^−/−^ and Plg-R_KT_^−/−^ mice was observed in parallel with decreased expression of AnxA1 and CD206, known to be involved in recognition and engulfment, respectively, of phagocytic material (38, 66). AnxA1 is one of many phagocytosis-related genes dysregulated in spleen and liver of Plg^−/−^ mice (22). Recently, we showed that treatment with or injection of Plg/Pla increases the efferocytic capacity of murine macrophages *in vitro* and *in vivo*, in an AnxA1-dependent manner (24). Plg and Pla increase AnxA1 expression in resolving macrophages, and AnxA1^−/−^ mice are refractory to Plg/Pla stimulation of efferocytosis, suggesting AnxA1 as a key molecule regulating Plg/Pla-mediated efferocytosis (24). Our current data implicating Plg-R_KT_^−/−^ in efferocytosis are in agreement with the requirement for cell surface lysine-binding sites for the phagocytosis-inducing effect of Plg/Pla (22). Thus, our results are consistent with a mechanism in which Pla bound to Plg-R_KT_ stimulates expression of AnxA1 and further efferocytosis. Here, we have shown that optimal induction of pSTAT3 and of IL-10 expression require both Plg/Pla and Plg-R_KT_, as an additional potential mechanism by which these molecules regulate efferocytosis. Indeed, it was recently shown that macrophage efferocytosis during inflammation resolution involves an IL-10 triggered STAT3 pathway (67). Taken together our results support the concept that Plg/Pla is required for macrophage reprogramming and efferocytosis, and these effects are dependent on Plg/Pla association with its receptor, Plg-R_KT_. Considering that these events are crucial steps of inflammation resolution, our results provide an important role for Plg/Pla and the receptor Plg-R_KT_ in the resolution of the inflammatory response.

## Data Availability

All datasets generated for this study are included in the manuscript and/or the Supplementary Files.

## Ethics Statement

All animal experiments were approved by the Institutional Animal Care and Use Committee of The Scripps Research Institute. Experiments using healthy volunteers were approved by the local research ethics committee (P/00/029 East London and The City Local Research Ethics Committee 1). Informed written consent was provided according to the Declaration of Helsinki.

## Author Contributions

JV, RP, LS, and LM designed research and analyzed data. JV, LS, and LM wrote the paper. JV, LS, MS, KL, GN-L, and NB performed experiments. MT and MP provided essential tools and expertise.

### Conflict of Interest Statement

The authors declare that the research was conducted in the absence of any commercial or financial relationships that could be construed as a potential conflict of interest.
