# Supplementary material for: Plasminogen and the Plasminogen Receptor, Plg-RKT, Regulate Macrophage Phenotypic, and Functional Changes
Source: Front Immunol. 2019 Jun 28;10:1458. doi: 10.3389/fimmu.2019.01458 (PMC6611080; doi:10.3389/fimmu.2019.01458)
Supplement: Supplementary file 1 [file Data_Sheet_1.PDF]

Supplementary Figure 1

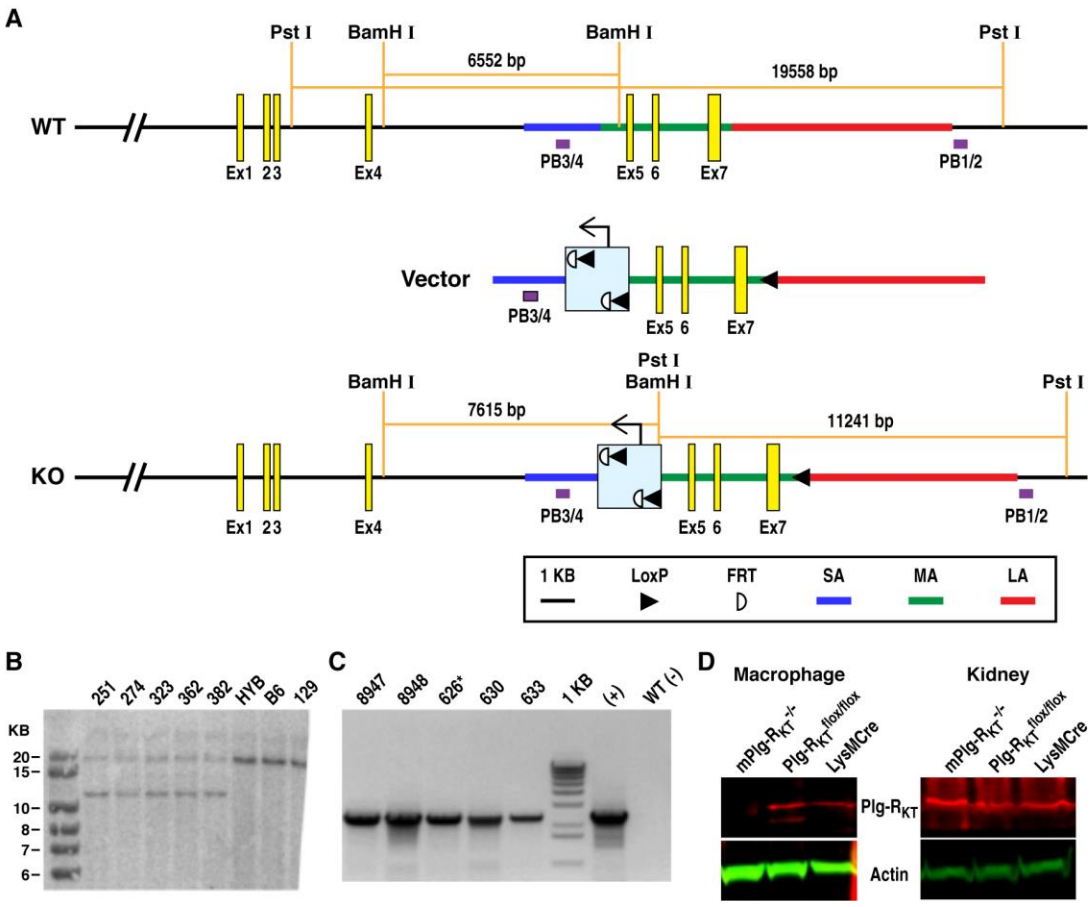

Figure S1. Generation of the Plg-R$_{KT}$*flox* allele and genetic characterization of Plg-R$_{KT}$$^{flox/flox}$ mice. Plg-R$_{KT}$*WT* targeting vector and the Plg-R$_{KT}$*flox* allele (A). ES cell clones carrying the Plg-R$_{KT}$$^{flox/neo}$ allele identified by Southern blotting (B). Germline-transmitting chimeric mice were mated to C57Bl/6 FLP mice to remove Neo cassette. PCR of tail DNA of resulting offspring shows integration and retention of distal LOXP site (C). Western blotting with anti-Plg-R$_{KT}$ and anti-actin in thioglycollate-elicited macrophages and kidney (D).

Supplementary Figure 2

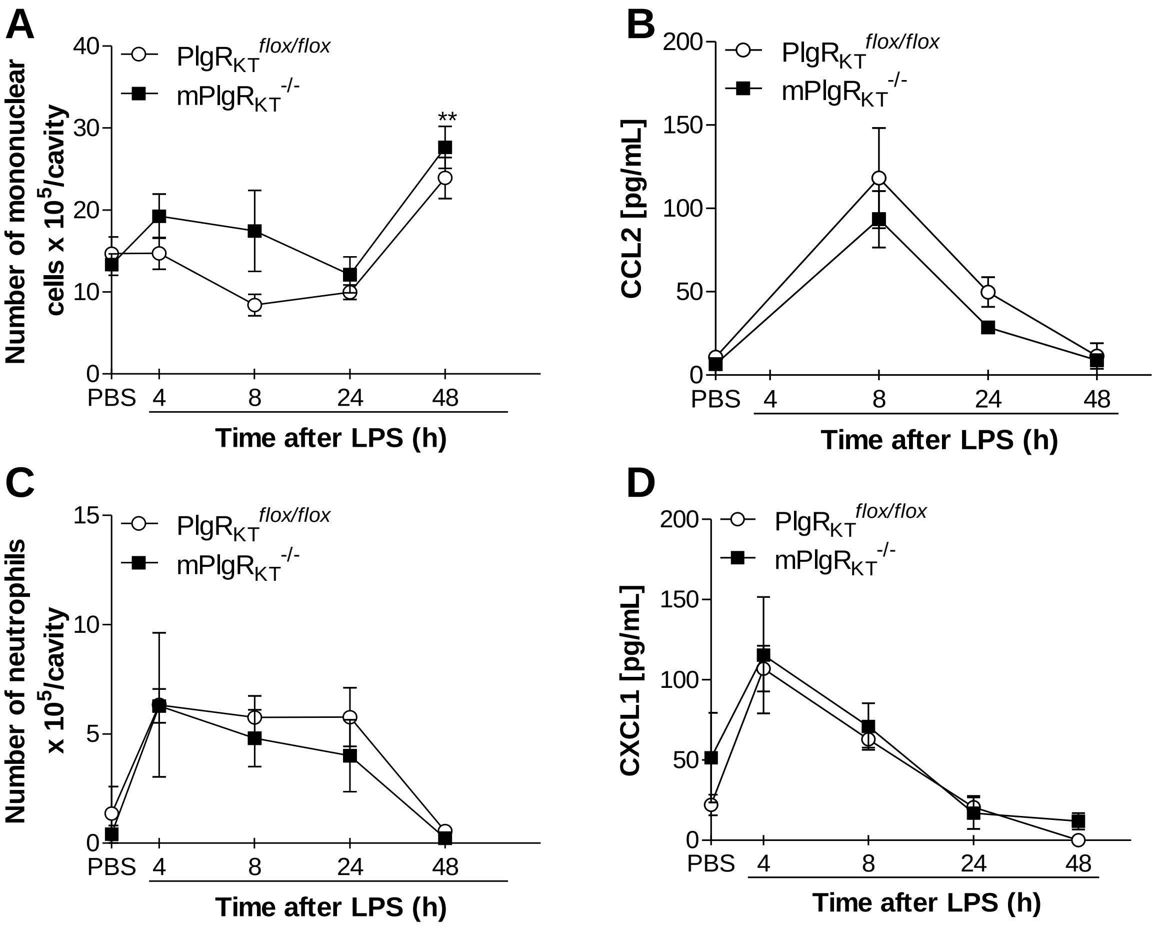

Figure S2. Kinetics of leukocyte infiltration during LPS-induced pleurisy in mice with the myeloid lineage specific deletion of Plg-R$_{KT}$. Plg-R$_{KT}^{flox/flox}$ and mPlg-R$_{KT}^{-/-}$ mice were injected with LPS (250 ng/cavity, i.pl.) or PBS. Cells present in the pleural cavity were harvested 4, 8, 24 and 48 hours after LPS challenge. The number of mononuclear cells (A), neutrophils (C) were evaluated by counting cytospin slides after stained with May-Grunwald-Giemsa. The levels of the monocyte chemoattractant CCL2 (B) and neutrophil chemoattractant CXCL1 (D) were evaluated from supernatants from pleural exudate by ELISA. Results are expressed as the number of cells per cavity and levels in pg/mL, and are shown as the mean ± SEM of at least five mice in each group. ** $P < 0.01$, when compared LPS-injected mice with PBS-injected mice.

Supplementary Figure 3

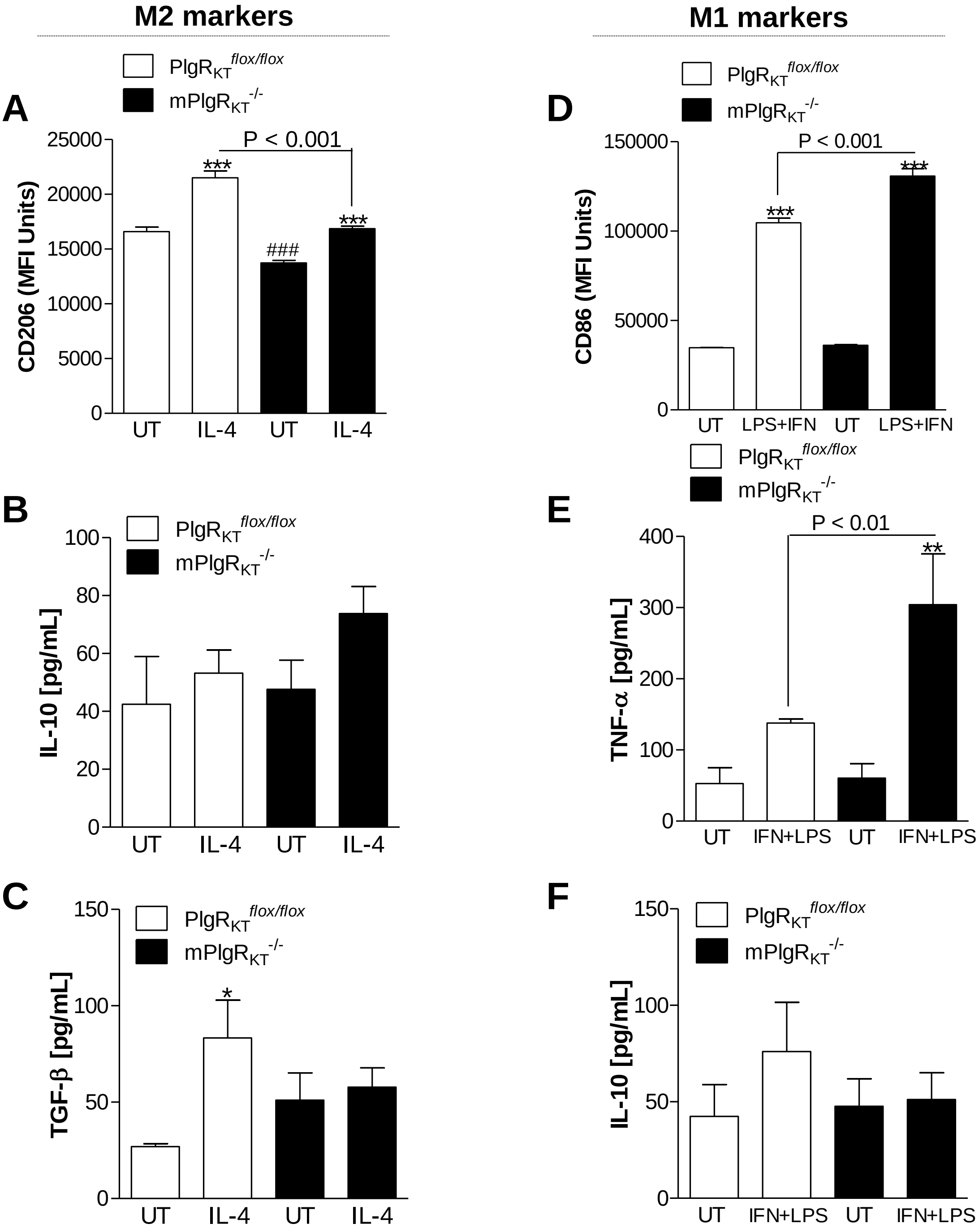

Figure S3. Effect of the myeloid lineage specific deletion of Plg-R_{KT} on macrophage polarization. BMDM from Plg-$R_{KT}^{flox/flox}$ and mPlg-$R_{KT}^{-/-}$ mice were washed 3 times with serum free media, and were either untreated or stimulated o with IL-4 (20ng/mL) or LPS (10ng/mL) + IFN (10ng/mL) for 24 hours. Then cells were analysed by flow cytometry for the expression of the M2 marker CD206 (A) and the M1 marker CD86 (D). The levels of secretory products of M2 macrophages IL-10 (B and F) and TGF-β (C), and of M1 macrophages TNF-α (E) were determined in supernatants by ELISA. Flow cytometric data and ELISA are expressed by MFI (mean fluorescence intensity) and levels in pg/mL, respectively, and are shown as the mean ± SEM. * $P < 0.05$, *** $P < 0.001$ when compared treated group with untreated (UT) BMDMs and ### $P < 0.001$ when compared UT Plg-$R_{KT}^{flox/flox}$ with UT mPlg-$R_{KT}^{-/-}$.

Supplementary Figure 4

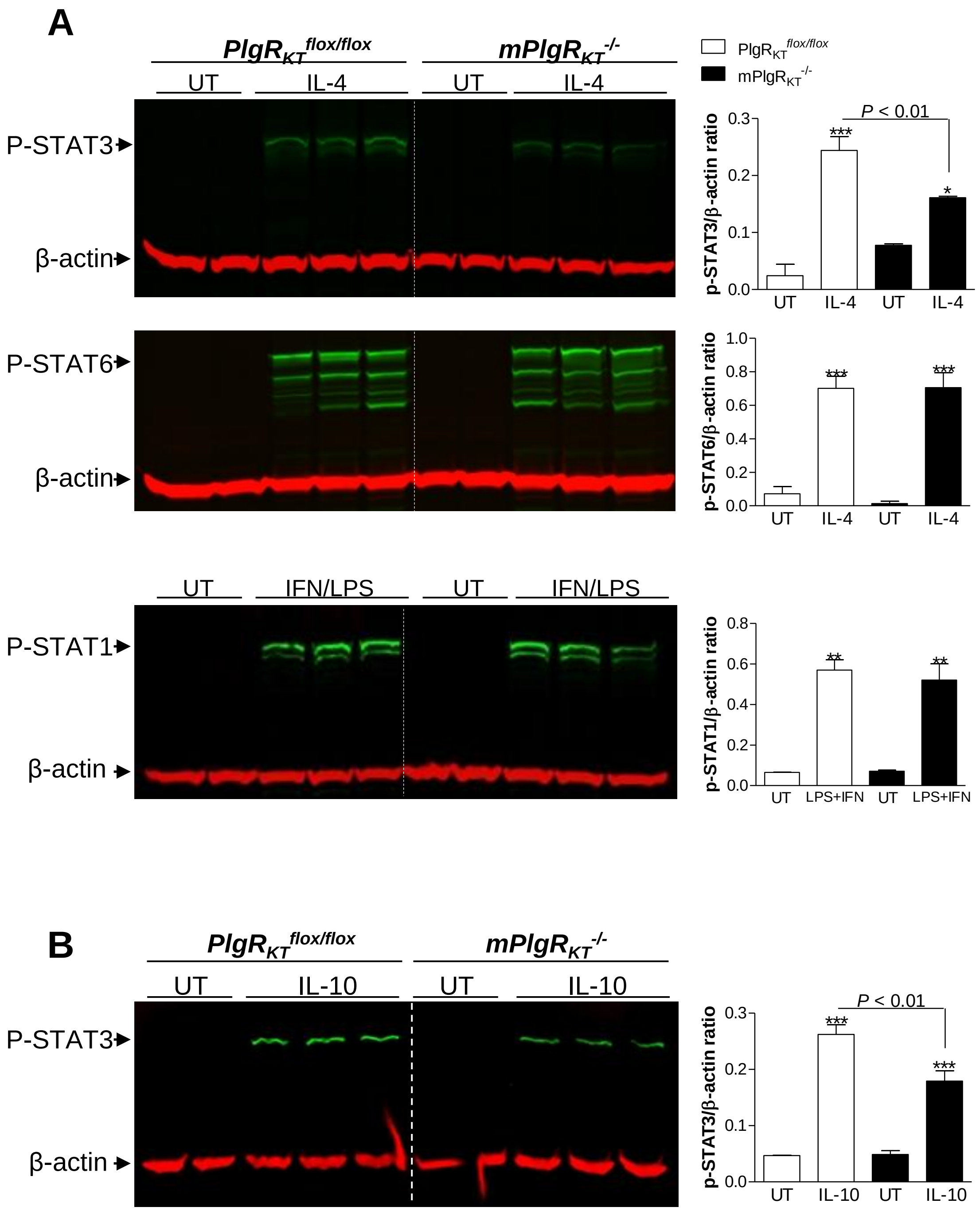

Figure S4. Effect of the myeloid lineage specific deletion of Plg-R$_{KT}$ on STAT signaling pathways. BMDMs from Plg-R$_{KT}^{flox/flox}$ and mPlg-R$_{KT}^{-/-}$ mice were washed 3 times with serum free media DMEM, and then treated with either IL-4 (20ng/mL), LPS (10ng/mL) + IFN (10ng/mL), IL-10 (20ng/mL) or untreated (UT) for 30 minutes (B-E). Cell lysates were electrophoresed and western blotted with antibodies against the indicated antigens. β-actin was used as a loading control. Densitometry analyses are shown on the right of each blot. * $P < 0.05$, ** $P < 0.01$, *** $P < 0.001$ when comparing treated with untreated (UT) BMDMs.

Supplementary Figure 5

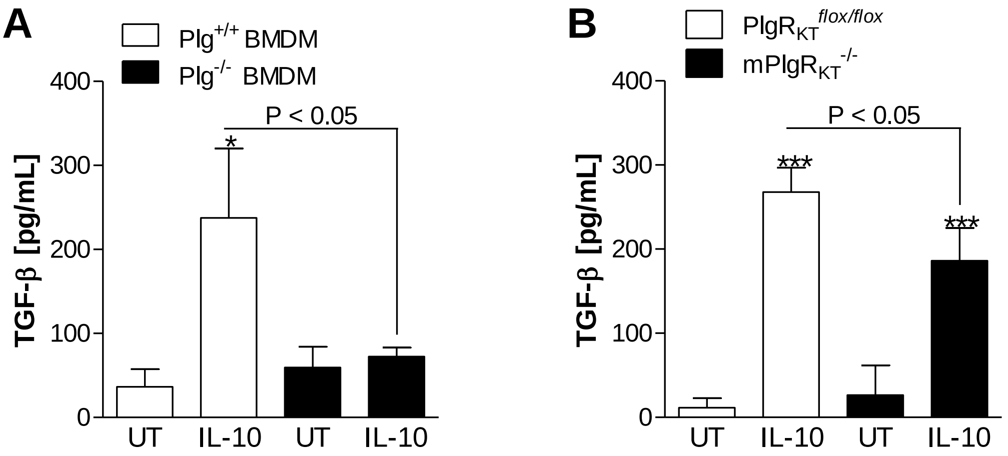

Figure S5. Effects of deletion of either Plg or Plg-R$_{KT}$ on TGF-β levels after macrophage polarization induced by IL-10. BMDMs from Plg$^{+/+}$, Plg$^{-/-}$, Plg-R$_{KT}^{flox/flox}$ and mPlg-R$_{KT}^{-/-}$ mice were separately washed 3 times with serum free media DMEM and then were either untreated (UT) or stimulated with IL-10 (20ng/mL) for 24 hours. The levels of secretory product of M2 macrophages TGF-β (A and B) were determined in conditioned media by ELISA. *$P < 0.05$, ***$P < 0.001$ when comparing treated with untreated (UT) BMDMs.

Supplemental Materials

List of primers used in this paper.

Gene	Forward sequence	Reverse sequence
Arginase-1	5'-TGACATCAACACTCCCCTGACAAC-3'	5'-GCCTTTTCTTCCTTCCCAGCAG-3'
Mannose receptor - CD206	5'-CATGAGGCTTCTCCTGCTTCTG-3'	5'-TTGCCGTCTGAACTGAGATGG-3'
iNOS	5’-AGCACTTTGGGTGACCACCAGGA-3’	5’-AGCTAAGTATTAGAGCGGCGGCA-3’
Gapdh	5'-AGAAGACTGTGGATGGCCCC-3'	5'-TGACCTTGCCCACAGCCTT-3'
